# Can acromegaly be controlled in all cases?

**DOI:** 10.1111/jne.70241

**Published:** 2026-08-05

**Authors:** Sabrina Chiloiro, Francesco Padovano Sorrentino, Alessandra Vicari, Antonella Giampietro, Pier Paolo Mattogno, Quintino Giorgio D'Alessandris, Rosalinda Calandrelli, Tommaso Tartaglione, Marco Gessi, Simona Gaudino, Laura De Marinis, Guido Rindi, Francesco Doglietto, Antonio Bianchi, Alfredo Pontecorvi

**Affiliations:** ^1^ Facoltà Di Medicina e Chirurgia Università Cattolica Del Sacro Cuore Rome Italy; ^2^ Division of Endocrinology, Metabolic Disease and Internal Medicine Fondazione Policlinico Universitario A. Gemelli IRCCS Rome Italy; ^3^ Dipartimento Di Neurochirurgia Fondazione Policlinico Universitario A. Gemelli, Istituto Di Ricovero E Cura a Carattere Scientifico (IRCCS) Rome Italy; ^4^ Dipartimento Di Scienze Radiologiche Fondazione Policlinico Universitario A. Gemelli, Istituto Di Ricovero E Cura a Carattere Scientifico (IRCCS) Rome Italy; ^5^ Unità di Anatomia Patologica, Dipartimento della donna e del bambino, e della salute pubblica Fondazione Policlinico Universitario A. Gemelli, Istituto di Ricovero e Cura a Carattere Scientifico (IRCCS) Rome Italy

**Keywords:** aggressive pituitary tumours, immune check point inhibitors, pasireotide, pegvisomant, target therapy

## Abstract

Acromegaly is a rare disease, due in most of the cases to a growth hormone (GH)‐secreting pituitary adenoma (PA), namely neuroendocrine tumour (PitNET). The treatment of patients with acromegaly is multimodal and multi‐step, including surgery, medical therapies, and radiotherapy. In the last 30 years, the therapeutic armamentarium for the treatment of acromegaly has progressively increased, and the therapeutic algorithm has been significantly modified through the identification of clinical, biochemical, and molecular biomarkers of treatment outcome. The personalization of acromegaly treatment has shifted the paradigm from a ‘trial‐and‐error’ to a ‘target‐to‐treat’ treatment approach to achieve early disease control and to reduce the risk of disease‐related comorbidities that may lead to an increased mortality. Nevertheless, despite the numerous improvements in the treatment of patients with acromegaly, disease control is not achieved in all patients, according to the results of randomized clinical trials, interventional studies, and prospective and retrospective observational studies. In parallel, the normalization of GH and IGF‐I levels may not be sufficient to control acromegaly related symptoms and prevent disease‐related comorbidities. In this review, we will report on the most recent aims of treatment and cure in patients with acromegaly, on the efficacy and predictors of response to conventional treatments (such as first‐ and second‐generation somatostatin receptor ligands and growth hormone receptor antagonist). A specific section will focus on the rarer aggressive disease pictures and on the treatment with systemic therapies (such as temozolomide and capecitabine) and on target therapies (such as neo‐angiogenesis and immune checkpoint inhibitors).

## 1 INTRODUCTION

Acromegaly is a rare disease caused by a growth hormone (GH)‐secreting pituitary adenoma (PA) or neuroendocrine tumour (PitNET). The prevalence of acromegaly is 5.9 per 100.000 inhabitants and the incidence is 0.38 cases per 100.000 inhabitants per year.^1^, ^2^ GH‐secreting PAs/PitNETs are heterogenous for clinical and biological behaviour, response to treatments and prognosis. Although most GH‐secreting PAs/PitNETs are invariably benign, around 25–45% of cases show local invasiveness and poor response to conventional treatments.^3^

The chronic excess of GH and insulin‐like growth factor 1 (IGF‐I), which is produced by the liver under GH stimulation, results in several systemic comorbidities, including cardiovascular, respiratory, metabolic, musculoskeletal, neurological, and neoplastic ones.^4^, ^5^

The presence of acromegaly related comorbidities and the persistence of high IGF‐I levels lead to an increased mortality risk, particularly for respiratory disease (19% of mortality causes), cardiovascular and cerebrovascular diseases (16% of mortality causes), and malignancies (14% of mortality causes).^6^, ^7^ Furthermore, older patients are at increased risk for mortality due to cardiovascular and cerebrovascular diseases.^7^ Therefore, the aims in the management of patients with acromegaly are to reach an early diagnosis, to effectively treat patients with a personalized and tailored approach, and to prevent, screen, and manage disease‐related comorbidities.^8^

The achievement of an early control of acromegaly (through surgery or medical therapies) is associated with improved survival outcomes.^9^ Indeed, a recent survival study conducted on 3569 patients with acromegaly from the UK register showed a higher survival rate in patients who achieved biochemical remission, with a strict correlation with the time‐to‐first remission: every one‐year increase in time to first remission correlated with a 1% reduction in survival rate.^9^

The treatment of patients with acromegaly is multimodal and multi‐step, including surgery, medical therapies, and radiotherapy.^3^, ^10^

The surgical resection of the adenoma remains the first‐line treatment in the large majority of patients, achieving disease cure/remission in approximately half of patients at referral centres.^11^ Surgical outcome remains directly influenced by the neurosurgeon's expertise and by the tumour pattern of growth and invasion, with lower chance of radical resection in large and invasive PAs/PitNETs.^11^

The medical treatment of acromegaly has undergone substantial changes in the past two decades, resulting in a substantial increase in the number of patients who have achieved disease control. A French cohort study including 275 patients demonstrated a higher rate of biochemical remission and a shorter time to remission in patients treated between 2010 and 2020 compared with those treated between 2000 and 2010, for an increased and widespread use of combination therapies and a multimodal approach.^12^

Moreover, a personalized treatment schedule was aim of investigation in recent years, to choose the most effective and safe treatment for an early disease control. The recent Spanish ACROFAST study proved that a personalized approach (including the evaluation of GH levels during octreotide test, the tumour expression of e‐cadherin and the tumour signal intensity in magnetic resonance image ‐MRI‐) allowed a higher number of patients to achieve acromegaly control in a shorter period of time, than the standard treatment protocol, shifting the paradigm from a “trial‐and‐error” to a “target‐to‐treat” approach.^13^

This review summarizes recent evidence on the efficacy of medical therapy in patients with acromegaly, predictors of response, rare aggressive disease phenotype, and the emerging target therapies.

## 2 DISEASE REMISSION: CLINICAL CRITERIA

To date, there are no precise standards for determining and defining the clinical disease burden in acromegaly.^14^ Therefore, a comprehensive clinical approach should include the assessment of acromegaly related symptoms and signs, the screening, diagnosis and management of comorbidities, both at acromegaly diagnosis and during follow‐up, regardless of biochemical control. Indeed, acromegaly related signs, symptoms and comorbidities may persist even in patients with biochemically controlled disease, affecting the quality of life and disease‐related mortality.^15^, ^16^

## 3 DISEASE REMISSION: BIOCHEMICAL CRITERIA

The definition of biochemical control has undergone numerous changes over the last 20 years, in terms of timing to test, IGF‐I and GH cut‐offs that were chosen to define disease control. Although the optimal assessment for post‐operative long‐term remission has yet to be defined, biochemical evaluation nowadays includes IGF‐I and basal GH.^3^ GH nadir levels during oral glucose tolerance test (OGTT) should be considered in specific clinical settings. According to the latest consensus, post‐surgery remission should be defined at least 3 months after surgery.^3^, ^11^, ^14^, ^17^, ^18^ Appropriate levels of IGF‐I are indicative of biochemical control and represent one of the main treatment goals in acromegaly.^11^, ^17^, ^18^ The early post‐surgical GH assessment (1–14 days after surgery) may also provide information on biochemical remission and on the degree of tumour resection.^19^, ^20^ The GH nadir during OGTT may predict long‐term remission, with different predictive values according to assay, nadir cut‐off points and the timing of post‐operative testing.^17^, ^21^, ^22^, ^23^, ^24^, ^25^ If OGTT is conducted 3 months after surgery, a GH nadir lower than 1 ng/mL is considered to reflect post‐operative remission. However, the cut‐off for GH nadir during OGTT is reduced to 0.4 ng/mL when modern ultra‐sensitive GH assays are used.

GH levels stabilize between the second and the fifth post‐surgery days, while IGF‐I levels can continue to decrease for up to 1–2 years after surgery.^24^, ^26^, ^27^ For patients treated with preoperative somatostatin receptor ligands (SRLs), IGF‐I levels should be tested after 3–6 months to evaluate surgical outcome.^3^ In the first year after surgery, IGF‐I levels should be evaluated every 3–6 months to confirm biochemical remission.^3^ Subsequently, IGF‐I measurements are recommended every 6–12 months. In patients with post‐surgery borderline IGF‐I levels, GH nadir during OGTT may provide additional information on biochemical remission.^3^

Additional considerations should be conducted for patients on medical therapy. To identify early response to injectable SRLs, IGF‐I levels should be assessed after 3–4 months since the first dose or dose titration.^6^ For patients on oral SRLs, evaluation of IGF‐I should be performed at least after 2 weeks of treatment.^28^ GH assessment is not informative and should not be performed for patients treated with medical therapy with GH receptor antagonist.^29^

## 4 DISEASE REMISSION: IMAGING CRITERIA

According to the most recent experts' consensus on acromegaly, postoperative imaging alone is not sufficient to define surgical outcome but should be integrated with clinical and biochemical parameters.^14^ The first postoperative pituitary MRI should be performed 3–6 months after surgery.^14^ Further pituitary MRIs should be performed during the follow‐up, particularly in patients with clinical and biochemical signs of active disease, or in the event of a change of treatments.^30^ Positron Emission Tomography‐computerized tomography (PET‐CT) using 11C‐L‐methionine (MET‐PET) could be an effective tool for detecting residual GH‐secreting adenomas after surgery, thereby enhancing the diagnostic power of MRI.

## 5 ACROMEGALY TREATMENTS AND EFFICACY

### 5.1 Surgery

The main objective of surgery is to selectively remove tumour tissue while identifying and preserving the normal pituitary gland. Surgery can lead to biochemical remission with rapid and complete normalization of GH levels and resolution of tumour‐induced mass effects, improving acromegaly related morbidity and mortality.^31^ The remission rate ranges from 75% to 90% of intrasellar and micro‐GH‐secreting Pas/PitNETs, and from 35% to 75% of macro‐GH‐secreting Pas/PitNETs.^32^, ^33^ High preoperative levels of GH and IGF‐I, larger tumour size, and invasion of the cavernous sinus are well recognised predictors of poor surgical remission.^32^, ^33^, ^34^

Repeated surgery may be considered in patients not responsive to medical therapies and with progression of residual disease. Remission rate at second surgery is lower than first surgery, ranging from around 15% (in cases of tumours with invasion of cavernous sinus) to around 27% (in not‐invasive tumours).^4^

Medical treatment with SRLs should be considered in patients at high anaesthesiologic risk and in those who refuse surgery.^14^ Several studies have confirmed that pre‐surgical SRLs treatment normalises GH and IGF‐I levels in 30–45% of patients after 6–12 months and induces significant tumour shrinkage in up to two‐thirds of patients after 1 year of treatment.^35^

Some studies have shown an improvement in typical acromegaly symptoms, such as obstructive sleep apnoea syndrome (OSAS), soft tissue swelling, left ventricular ejection fraction (LVEF), and systolic and diastolic blood pressure in patients treated with SRLs for at least 3–6 months before surgery.^36^, ^37^ Presurgical medical therapy may facilitate anaesthesiologic procedures by reducing the swelling and oedema of the soft tissue of the oropharynx, hypopharynx, tongue, and larynx. Therefore, patients with acromegaly related symptoms, cardiovascular disorders, and OSAS may benefit from 3 to 6 months of presurgical medical therapy with SRLs to improve anaesthesiologic management.^37^

### 5.2 Medical treatment

#### 5.2.1 First generation SRLs

SRLs remain the first‐line medical therapy for acromegaly. Injectable first‐generation (fg)‐SRLs, such as octreotide long‐acting release (LAR) and lanreotide, have been used in clinical practice for over 30 years. Several randomized clinical trials (RCTs), prospective and retrospective real‐life studies, have provided favourable data on their efficacy.

Through binding to somatostatin receptors (SSTRs), SRLs reduce GH secretion and cell proliferation and increase cell apoptosis.^38^

The fg‐SRLs had the greatest affinity for subtype 2 of the SSTRs (SSTR2), with a binding affinity of 0.38 + 0.08 nmol/L for half‐maximal effective concentration of octreotide and of 0.54 + 0.08 nmol/L for lanreotide.^39^ Efficacy of octreotide LAR and lanreotide is quite similar, achieving disease control in up to 45–55% of patients.^40^, ^41^

As reported in Table 1, several factors were identified as positive predictors for response to fg‐SRLs, such as female gender, older age, smaller tumour dimension, intrasellar extension, hypointense tumour signal in T2‐weighted MRIs.^42^, ^43^, ^44^, ^45^, ^46^ Moreover, extensive pathology investigations were historically conducted to predict fg‐SRLs outcome, identifying as the more significant the cytokeratin granulation pattern, the Ki‐67 immunolabeling, and the expression of E‐cadherin, beta‐arrestin, ZAC1, RKIP, and AIP.^47^, ^48^, ^49^, ^50^, ^51^ Several studies proved also that the high number of tumour cells positive for the SSTR2 and the cell membranous expression of the SSTR2A are associated with a better response to fg‐SRLs.^46^, ^52^ Moreover, recent studies on tumour microenvironment (TME) showed novel knowledge on potential predictors of tumour behaviour and efficacy of fg‐SRLs, providing that a higher tumour infiltering CD8+ lymphocytes^53^ was associated with a good response to fg‐SRLs and that a higher CD8+/CD68+ ratio was associated with a good response to fg‐SRLs in patients naïve to treatment with fg‐SRLs before surgery.^54^

**TABLE 1:** Predictive factors for a good response to medical therapy.

Therapy	Parameters	Predictors of good response
fg‐SRLs	Clinical	Female gender Older age
	Radiological	Smaller tumour volume Intrasellar extension Radiological hypointensity in T2‐weighted MRI scans
	Molecular	Densely granulated pattern Higher SSTR2 expression (>membranous expression of SSTR2A) ZAC1 expression E‐caderin expression Lower beta‐arrestin expression Negative RKIP and AIP expression Low Ki‐67.
	Tumour microenviroment	Higher tumour infiltering CD8+ lymphocytes Higher tumour infiltering CD8+/CD68+ ratio (in patients naïve to treatment with fg‐SRLs before surgery).
Dopamine agonists	Biochemical	Baseline levels of IGF‐1 <1.5× ULN Higher PRL levels
Pasireotide LAR	Clinical	Partial biochemical response to fg‐SRLs
	Biochemical	Baseline levels of IGF‐1 <2.3× ULN
	Radiological	Less invasive tumours (Knosp grades 1–2) Radiological hyperintensity in T2‐weighted MRI scans
	Molecular	The presence of the fl‐GH receptor isoform Sparsely granulated pattern Membranous expression of SSTR2A, SSTR5 expression.
Pegvisomant	Biochemical	Baseline levels of IGF‐1 <3.3× ULN
	Radiological	Less invasive tumours (Knosp grades 1–2)
	Molecular	Low ki‐67 (<4%)
Pasireotide LAR + Pegvisomant	No available data	

The 2022 World Health Organization (WHO) classification of Endocrine and Neuroendocrine Tumours identified specific subtypes of pituitary neoplasia characterized by a poor response to fg‐SRLs, such as the sparsely granulated somatotroph tumours, the immature PIT1‐lineage, and acidophil stem cell PitNETs.^55^ Sparsely granulated somatotropinomas were hypothesized to be characterized by a lower SSTR2 expression, resulting in a poor response to SSTR2‐targeting medical therapy.^56^, ^57^, ^58^, ^59^, ^60^

If available, oral octreotide capsules can be used as an alternative to injectable fg‐SRLs, as studies have demonstrated improved compliance while maintaining similar efficacy.^61^

Modified fg‐SRLs administration schedules, such as high dose (HD) of fg‐SRLs or shorter frequency (SF) of administration, may further reduce IGF‐I by around 30%, at least in patients who had a partial response to the standard dosing regimen.^62^, ^63^, ^64^ Evidence on predictors of response to HD/SF fg‐SRLs administration is limited. A single study identified the IGF‐I levels reached at 6 months of standard dose of fg‐SRLs as a predictor of response to SF administration, as lower levels of IGF‐I correlate with a better response.^65^ Conversely, dose administration intervals can also be extended beyond the standard 4 weeks in patients considered responsive to standard dose of fg‐SRLs, to maintain biochemical control while reducing adverse effects.^66^, ^67^

#### 5.2.2 Dopamine agonists

Cabergoline is the most effective dopamine 2 receptor (D2) agonist. The use of Dopamine agonists (DA) in acromegaly is supported by evidence of the expression of D2 receptors in GH‐secreting PAs/PitNETs.^68^

DA is rarely used as monotherapy in patients with acromegaly, achieving disease control in less than one third of patients, with an efficacy lower than those achieved by other medical therapies.^69^ Some studies found that baseline levels of IGF‐I lower than 1.5× upper limit of normality (ULN) can predict response to cabergoline (Table 1).^14^, ^69^ The role of high prolactin (PRL) levels as a predictive factor remains debated.^69^ The combination treatment cabergoline plus fg‐SRLs is also a viable option but remains limited to patients with mildly elevated IGF‐I levels (<2× ULN) during monotherapy with fg‐SRLs.^69^

#### 5.2.3 Pasireotide LAR

Pasireotide is a multiligand injectable SRL, with the greatest binding affinity for subtype 5 of the SSTR (SSTR5). The binding affinity for SSTR5 is 0.16 + 0.01 nmol/L for the half‐maximal effective concentration of Pasireotide, for SSTR2 is 1.0 + 0.1 nmol/L for the half‐maximal effective concentration of Pasireotide, for SSTR3 is 1.5 + 0.3 nmol/L for the half‐maximal effective concentration of Pasireotide, for SSTR1 is 9.3 + 0.1 nmol/L for the half‐maximal effective concentration of Pasireotide, and for SSTR4 is higher than 100 nmol/L for the half‐maximal effective concentration of Pasireotide.^39^

Pasireotide Lar is approved as a second‐ or third‐line treatment in patients not responsive to fg‐SRLs. Data on the efficacy of Pasireotide Lar derived mainly from clinical trials. In patients with absent or partial response to fg‐SRLs, Pasireotide Lar achieved disease control in 20% of cases.^70^, ^71^, ^72^ In real‐world clinical studies, the remission rate significantly varies, reaching 93% in a selected cohort of patients.^16^, ^73^, ^74^, ^75^ A recent systematic review of real‐world data on Pasireotide Lar reported a remission rate of 60%.^76^ Pasireotide Lar is also associated with more significant tumour shrinkage in up to 40% of patients, compared to fg‐SRLs.^77^

Treatment with Pasireotide Lar is also associated with reduced central adiposity^78^ and overall neutral effect on dyslipidaemia, except for a modest raise in high‐density lipoprotein cholesterol (HDL‐C).^79^ Several studies reported a higher incidence of hyperglycaemia and impaired glucose tolerance, and an approximately 3‐fold increased risk of developing diabetes mellitus during treatment with Pasireotide Lar.^80^, ^81^

In patients who have attained biochemical disease control, lowering the dosage of pasireotide lar may improve the glycaemic profile while preserving disease control, according to a recent real‐world study.^82^

However, this treatment regimen requires consistent monitoring of IGF‐I levels to exclude treatment‐induced escape, a phenomenon that is more prevalent in patients who have achieved a biochemical response to Pasireotide at a later stage (after 20 months of treatment).^82^, ^83^

Predictive factors for a good response to Pasiretoide LAR include lower pre‐treatment levels of IGF‐I (<2.3× ULN), partial response to previous treatment with fg‐SRLs, non‐invasive tumour growth (Knosp grades 1–2), hyperintense tumour signal in T2‐weighted MRI scans, and some specific tumour pathological features, such as the cytokeratin sparsely granulated pattern and the membranous expression of the SSTR2A and SSTR5 in the tumour cells, as summarized in Table 1.^52^, ^84^, ^85^, ^86^, ^87^, ^88^, ^89^, ^90^

#### 5.2.4 Pegvisomant

Pegvisomant is a GH receptor antagonist that competes with GH for binding to its receptor, thereby decreasing IGF‐I synthesis and secretion from the liver. In clinical trials, pegvisomant achieved biochemical control in up to 92% of patients when used as a first‐line therapy,^91^ and in around 63–90% of patients as second‐ or third‐line therapy.^92^, ^93^, ^94^

Treatment with Pegvisomant may be scheduled as monotherapy or in combination with SRLs or dopamine agonist, reflecting significant differences in disease phenotypes. As reported in an Italian multicentre study, patients with higher levels of GH and IGF‐I and carrying large tumour residual are more likely to be treated with the combination Pegvisomant + fg‐SRL than with Pegvisomant in monotherapy, reflecting the predisposition of physicians to choose combination therapy in patients with suspicion of more aggressive acromegaly.^88^ Pegvisomant in monotherapy instead was historically more often prescribed to elderly patients, with small tumour residual, far from the optical chiasm. However, more recently, successful treatment with Pegvisomant in monotherapy was reported in young patients affected by acro‐gigantism due to AIP‐mutated large and invasive somatotroph tumours and in pediatric patients affected by X‐linked acrogigantism, due to duplication of GPR101.^95^, ^96^

In clinical studies, the combination cabergoline plus pegvisomant achieved remission in a further 28% of patients who were not previously controlled with pegvisomant in monotherapy.^97^ However, there are very few clinical studies on this combination, making it difficult to assess long‐term efficacy. Nevertheless, the combination pegvisomant plus cabergoline may represent a treatment option for patients who are intolerant to fg‐SRLs.^14^

Data on efficacy of Pegvisomant are influenced by issues with dose adjustment. As reported in Acrostudy analysis, conducted on 2221 patients, the mean daily dose of Pegvisomant was similar among patients who achieve and who did not achieve the biochemical control.^92^, ^98^ Several studies investigated the potential predictors of need for higher dose of Pegvisomant to normalize IGF‐I levels, identifying among the most relevant the female gender, the higher weight, the higher GH and IGF‐I levels before starting treatment with Pegvisomant, the presence of diabetes mellitus and the concomitant treatment with insulin.^99^, ^100^, ^101^, ^102^

The poor response to Pegvisomant treatment is limited to cases of aggressive acromegaly disease. Predictive factors of poor response to the combination Pegvisomant plus fg‐SRLs therapy are higher baseline levels of IGF‐I (>3.3× ULN), invasive tumour growth (Knosp grade 3–4), and highly proliferative tumours (with high Ki67 immunolabeling >4%), as summarized in Table 1.^84^, ^88^, ^92^

The investigation of Pegvisomant effects on residual tumour mass was one of the main objectives of ACROSTUDY, providing a quite neutral effect.^92^ Further studies suggested that tumour regrowth during treatment with Pegvisomant may reflect a more aggressive tumour behaviour and phenotype, rather than an effect of the drug. Tumour residual regrowth was in fact reported in patients with higher levels of GH and IGF‐I before starting treatment with Pegvisomant and in patients with large tumour residual.^88^

Treatment with Pegvisomant has been reported to improve glucose tolerance in patients with acromegaly, even in cases with biochemical active disease.^103^ Instead, long‐term side effects of Pegvisomant are rare and include hypertrophy of subcutaneous and visceral fat, particularly at the injection site.^104^

### 5.3 Radiotherapy

Radiotherapy remains a viable treatment option for patients not responsive to medical therapies, or not eligible for surgery due to the persistence of inoperable residual disease (e.g., invasion of the cavernous sinus).

Stereotactic radiotherapy achieves biochemical control and reduces tumour mass in up to 60% of patients.^105^, ^106^, ^107^ However, response to radiotherapy can take up to 10 years to reach its maximum effect.^105^, ^106^, ^107^

## 6 AGGRESSIVE PITUITARY TUMOURS: DEFINITION AND PREDICTORS

Although most PAs/PitNETs are characterized by a benign behaviour and are effectively managed with surgery or medical treatment, a small percentage of PAs/PitNETs exhibit aggressive behaviour, with multiple recurrences and progression.

In 2015, Cuevas‐Ramos et al. proposed a structural and functional classification of acromegaly, analysing data collected in the Cedars‐Sinai registry from 1178 patients.^108^ This classification identified three subtypes of disease. The subtype 1 included around 50% of cases and encompassed mainly older patients, with low GH tumoural secretion, non‐aggressive and non‐proliferating micro‐ or macro‐adenomas, with a frequent concave picture (due to the prevalent extension to the sphenoid sinus rather than to the suprasellar region), abundant immunoreactive for GH, p21 and SSTR2, and with a densely granulated cytokeratin pattern. Disease outcome was mostly favourable, requiring typically a single surgical resection and medical treatment with two or fewer classes of drugs. The subtype 2 included around 19% of patients and encompassed mainly cases with intermediate GH tumoural secretion, non‐invasive, non‐proliferating or low‐proliferating macroadenomas, without significant tumour extension and without invasive growth (flat tumour image), weakly immunoreactive for GH, p21 and SSTR2, and densely or sparsely granulated cytokeratin patterns. Disease outcome was intermediate, requiring typically one or two surgical resections and medical treatment with two or less classes of drugs. The subtype 3 included around 31% of patients and encompassed mainly cases with high GH tumoural secretion, aggressive and invasive macro‐adenomas, with a frequent “peanut” picture (due to the extension to both the sphenoid sinus and suprasellar regions with commonly encountered optic chiasm compression), with low immunoreactive for GH, p21 and SSTR2, and a sparsely granulated cytokeratin pattern. Disease outcome was mostly adverse and poor, requiring typically two or more surgical resections and medical treatment with two or more classes of drugs.

More recently, the PANOMEN‐3 classification was reported to predict the prognosis of PA/PitNETs. Evidence of the ability of the PANOMEN‐3 classification in predicting the outcome of patients with acromegaly is still lacking, as studies were conducted of a heterogenous patient's cohort including several types of PAs/PitNETs.^109^, ^110^, ^111^, ^112^, ^113^, ^114^

Although the most recent classifications of PAs/PitNETs have attempted to identify aggressive tumours, a univocal classification is not yet available. In fact, the identification of an aggressive tumour can only be established during follow‐up, through observation of the growth rate and response to treatments.^73^ The median time from diagnosis of PA/PitNET to the detection of aggressive behaviour is 5.5 years, while the maximum reported time is 33 years.^115^

**FIGURE 1:**
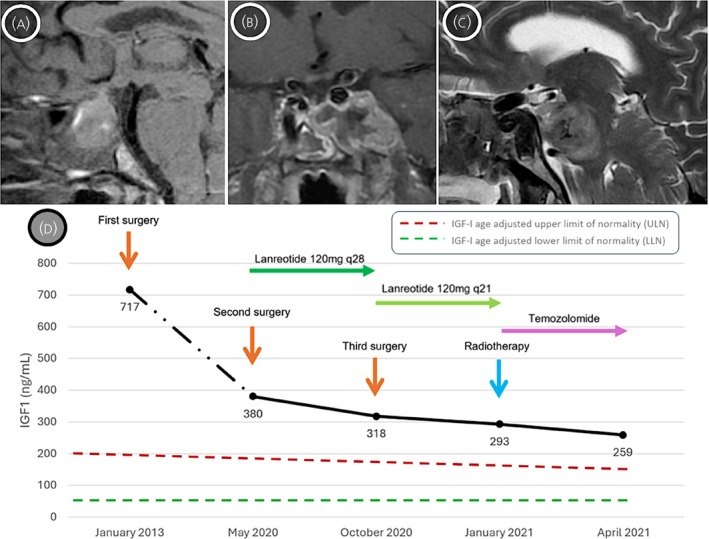
(A) Brain and pituitary MRIs showing a 30 mm GH‐secreting PAs/PitNETs with invasion of the left cavernous sinus at diagnosis of acromegaly (T1‐weighted post gadolinium sagittal MRI), (B) Brain and pituitary MRIs showing a PA/PitNETs residual disease in progression of 48 mm with invasion of left cavernous sinus after surgery and during treatment with fg‐SRLs (T1‐weighted coronal MRI), (C) Brain and pituitary MRIs showing further progression of the tumour residual disease, resulting in contact with the inferior wall of the terminal tract of the internal carotid artery and with the left optic nerve, with encasement of the intra‐cavernous tract of the internal carotid artery, invasion of the Meckel's cavity and prepontine cistern, compression on the pons, encasement of the left superior cerebellar artery (T2‐weighted sagittal MRI); (D) Representative scheme of the treatment history and biochemical outcome of the patient.

The European Society of Endocrinology (ESE) recommended (in last clinical practice guidelines) to diagnose an aggressive pituitary tumour (APTs) in patients with invasive PAs/PitNET and either (1) unusually rapid tumour progression or (2) clinically relevant tumour progression despite optimal standard therapies, including surgery, radiotherapy and conventional medical treatments.^74^ The Figure 1 showed the MRIs of a GH secreting APT at diagnosis (A) and during follow‐up (B and C) for a clinically relevant and unusually rapid tumour progression, despite optimal, multimodal and subsequent treatments (Figure 1D).

**FIGURE 2:**
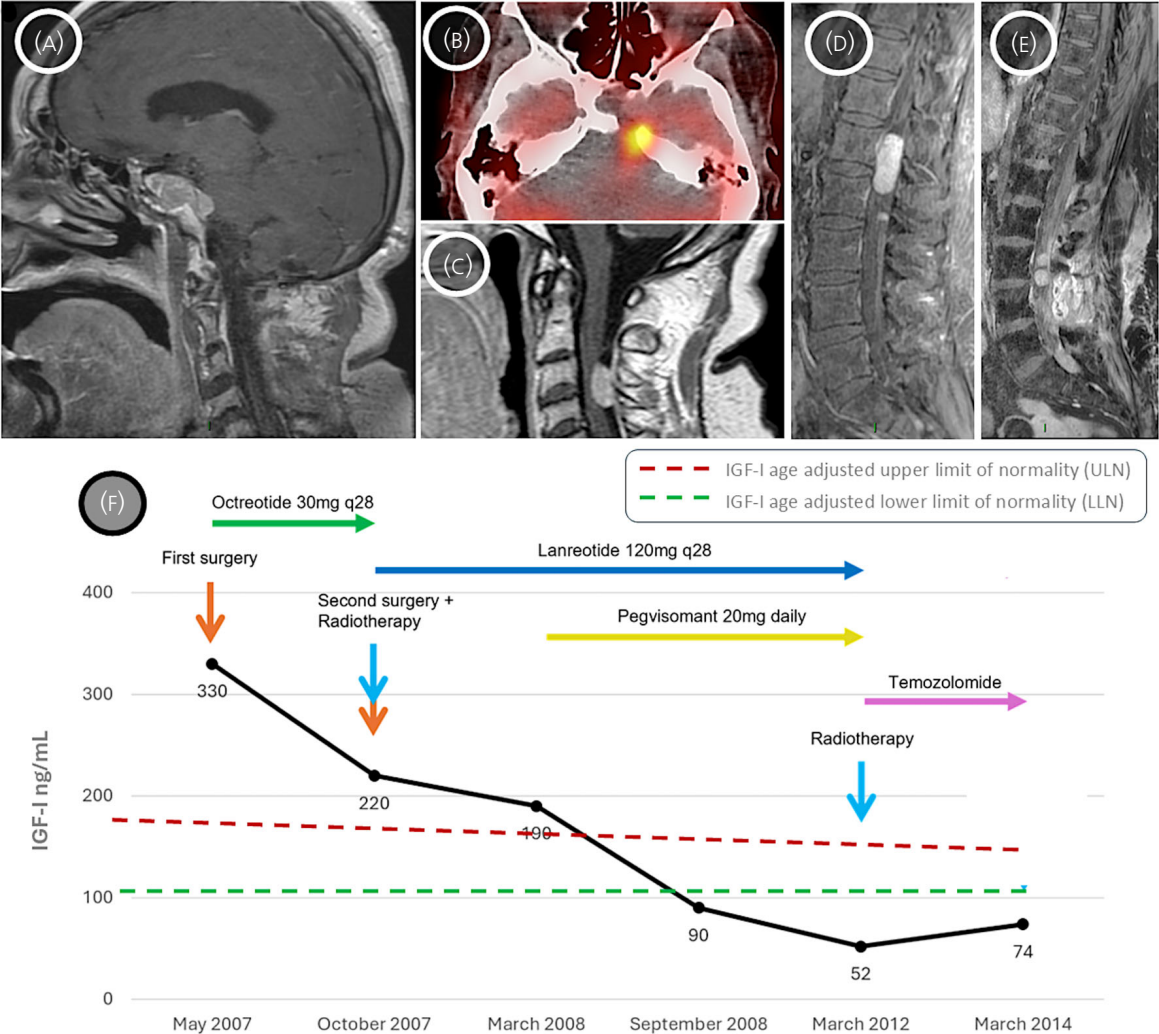
(A) Brain and pituitary MRIs showing residual GH‐secreting APT (sagittal post gadolinium administration T1‐weighted MRI) with intense uptake of 18‐fluorodeoxyglucose (18‐FDG) tracer at positron emission tomography (PET) at left cavernous sinuses (B); (C) cervical, (D and F) lumbar and sacral spinal cord metastatic dissemination (C: Sagittal post gadolinium administration T1‐weighted MRI, d: Sagittal post gadolinium administration T1‐weighted MRI and e: Sagittal T2‐weighted MRI); (F): Representative scheme of the treatment history and biochemical outcome of the patient.

Approximately 0.5% of all PAs/PitNETs exhibit aggressive behaviour, and fewer than 0.1% will become malignant, with the development of metastasis.^75^ The Figure 2 showed the MRIs of a residual GH secreting APT (A) with intense uptake of 18‐fluorodeoxyglucose (FDG) tracer at positron emission tomography (PET) at left cavernous sines (B), with metastatic dissemination at cervical (C) and lumbar and sacral spinal cord (D and E), despite optimal, multimodal and subsequent treatments (F).

The prompt identification of aggressive pituitary tumours is crucial for the increased morbidity and mortality, even in the absence of metastases.^74^

Although even small and not‐invasive PAs/PitNETs with benign/indolent histology features at diagnosis may develop an aggressive and metastatic phenotype during follow‐up,^116^ proliferative markers (such as high Ki67, p53 expression and a high mitotic count) may predicts an aggressive behaviour.^117^, ^118^, ^119^ Liu et al. demonstrated that a tumoural EGFR overexpression and a monthly tumour growth rate >2.2% are independent predictors of aggressive behaviour.^120^ Consequently, the last ESE guidelines on management of aggressive pituitary tumours and pituitary carcinomas recommend including proliferation markers (mitotic count, ki67 or MIB1, and p53) in pathological and immunohistochemical assessment for PAs/PitNETs, together with the standard stains for pituitary hormones and pituitary‐specific transcription factors.^74^

## 7 AGGRESSIVE PITUITARY TUMOURS: TREATMENTS

The treatment for aggressive PAs/PitNETs requires multidisciplinary evaluation and is often based on multimodal and multi‐step treatments.

The first‐line treatment for APTs is mainly based on temozolomide (TMZ).^74^ As an oral alkylating agent, TMZ exerts its cytotoxic effect by methylating DNA, which ultimately triggers tumour cell apoptosis. The efficacy of TMZ ranges from 20 to 70% of cases, resulting in more successful outcomes in early treated patients.^121^ According to ESE clinical practice guidelines for the management of APTs and PCs, the efficacy of temozolomide should be evaluated according to RECIST criteria.^74^ For patients with APTs/PCs in progression during treatment with TMZ, multidisciplinary discussion should orient the treatment according to tumour molecular profiling and patients' needs, as it relieves mass effect.^74^

Tumour molecular profiling is crucial to personalize treatments in APTs and PCs before candidate patients are targeted for therapies such as peptide receptor radionuclide therapy (PRRT), immunotherapy, angiogenesis inhibitors, or capecitabine and temozolomide combination (CAPTEM protocol).

According to the latest ESE guidelines, patients with APTs and PCs may be considered firstly for clinical trials with immune checkpoint inhibitors (ICIs).^74^ ICIs have indeed been shown to be safe and feasible in patients with APTs and PCs, with higher efficacy in tumours with mismatch repair deficiency (MMRd) or a high mutational burden.^122^ Lastly, participation in clinical trials with targeted agents (such as tyrosine kinase inhibitors) or cytotoxic chemotherapy is encouraged by ESE guidelines.^74^ The growing interest in the TME has revealed promising results also from anti‐angiogenic drugs, particularly bevacizumab: around 57% of patients receiving bevacizumab monotherapy may achieve a stable disease.^123^

## 8 ACROMEGALY DUE TO AGGRESSIVE PITUITARY TUMOURS: OPTIONS OF TREATMENT

The treatment of patients with acromegaly due to APTs and PCs remains an open issue, including target therapies but also the combination of GH/IGF‐I lowering treatments. The combination of Pasireotide Lar plus Pegvisomant has been reported in patients with aggressive acromegaly.^124^ To date, the combination of Pasireotide Lar and Pegvisomant is the most effective medical therapy for acromegaly, although in the absence of predictors of response (Table 1).^124^

The latest Consensus Statement recommends this combination as a third‐line therapy for multi‐drug resistant acromegaly, suggesting its use in patients with inadequate biochemical or disease control despite optimised second‐line therapy with fg‐SRL, Pegvisomant or Pasireotide Lar.^14^

A recent monocentric study has also described this combination as effective therapy for preventing occurrence of fragility fractures, in the clinical setting of aggressive acromegaly.^125^ However, given the metabolic adverse effects of Pasireotide, this treatment requires careful evaluation in patients with impaired glucose tolerance.^126^

Data on target therapies in GH‐secreting APTs and PCs remain limited to small case series and case reports: until now a single case of a patient with acromegaly and treated with ICI was reported, achieving a stable disease.^122^ Similarly, a single case of an acromegaly patient with APT treated with bevacizumab was reported in the ESE survey, reaching a partial response.^123^ Moreover, bevacizumab has been used as part of a multimodal approach alongside temozolomide, octreotide, and radiotherapy in a childhood‐onset, AIP‐mutated, GH‐secreting APT, achieving a stable disease.^127^ Regarding cytotoxic chemotherapy, the combination of TMZ and capecitabine (CAPTEM) has been reported in a single patient with acromegaly, achieving a partial response.^128^

## 9 WHY CAN ACROMEGALY NOT BE CONTROLLED IN ALL CASES?

As outlined in this review, the armamentarium for treating acromegaly has expanded over the last 30 years. Despite the high efficacy of acromegaly therapies, none of the currently available drugs have achieved disease control in all cases, considering various study designs (interventional studies, randomized clinical trials, and prospective and retrospective observational studies).

The improvement of understanding on the molecular profile of somatotropinomas, as well as of drug pharmacokinetics and pharmacodynamics, has facilitated the personalization of treatment schedule in patients with acromegaly, thereby enhancing therapeutic efficacy and safety. Regrettably, a 100% control rate was not achieved, even in the context of personalized therapy.^13^

This outcome can be influenced by a variety of factors, such as the availability of drugs in various countries worldwide, cost–benefit assessments, the necessity of selecting a therapy based on patient adherence and concomitant pathologies, and, most importantly, the aggressiveness of the tumour.^128^

Although very rare, tumours with aggressive biological behaviour, not responsive to conventional therapies, even if used through personalized, combined, and multimodal regimens, have been reported. For these rare cases, systemic target therapies with temozolomide, capecitabine, ICIs, and anti‐VEGF monoclonal antibodies have been reported; however, because these reports are limited to small case series or case reports, there is no conclusive evidence of treatment effectiveness (mostly stable disease or partial response).

## 10 CONCLUSION

The therapeutic arsenal for the treatment of acromegaly has been expanded, enabling the management of acromegalic disease in a growing number of patients. The traditional approach to acromegaly therapy management has undergone a substantial transformation, transitioning from the objective of reducing IGF‐I levels to the comprehensive management of the disease, which encompasses the enhancement of acromegaly related comorbidities and biochemical and radiological outcomes. Furthermore, the identification of clinical, biochemical, and molecular predictors of treatment response has facilitated the development of patient‐specific and personalized treatment regimens, which may achieve early disease control. The poor response to GH/IGF‐I lowering drugs (in monotherapy or in combination) is restricted to rare cases of aggressive phenotype of acromegaly in the context of optimal clinical management and personalized treatment.

## AUTHOR CONTRIBUTIONS

**Francesco Padovano Sorrentino:** Conceptualization; investigation; writing – original draft; writing – review and editing; data curation; methodology; validation; visualization. **Alessandra Vicari:** Conceptualization; investigation; writing – original draft; writing – review and editing; data curation; methodology; validation; visualization. **Pier Paolo Mattogno:** Validation. **Quintino Giorgio D'Alessandris:** Validation. **Tommaso Tartaglione:** Methodology; supervision. **Rosalinda Calandrelli:** Validation. **Antonella Giampietro:** Data curation; methodology. **Simona Gaudino:** Methodology; supervision. **Antonio Bianchi:** Methodology; validation; supervision. **Sabrina Chiloiro:** Conceptualization; methodology; data curation; investigation; writing – original draft; writing – review and editing; supervision. **Marco Gessi:** Methodology; supervision. **Guido Rindi:** Methodology; supervision. **Alfredo Pontecorvi:** Methodology; validation; supervision. **Francesco Doglietto:** Methodology; supervision; visualization. **Laura De Marinis:** Methodology; supervision.

## CONFLICT OF INTEREST STATEMENT

The authors declare that the research was conducted in the absence of any commercial or financial relationships that could be construed as a potential conflict of interest.

## Data Availability

Data sharing not applicable to this article as no datasets were generated or analysed during the current study.
